# A comprehensive systematic review dataset is a rich resource for training and evaluation of AI systems for title and abstract screening

**DOI:** 10.1017/rsm.2025.1

**Published:** 2025-03-07

**Authors:** Gary C. K. Chan, Estrid He, Janni Leung, Karin Verspoor

**Affiliations:** 1 School of Computing Technologies, RMIT University, Melbourne, VIC, Australia; 2 National Centre for Youth Substance Use Research, University of Queensland, Brisbane, QLD, Australia; 3 School of Computing and Information Systems, The University of Melbourne, Melbourne, VIC, Australia

## Abstract

When conducting a systematic review, screening the vast body of literature to identify the small set of relevant studies is a labour-intensive and error-prone process. Although there is an increasing number of fully automated tools for screening, their performance is suboptimal and varies substantially across review topic areas. Many of these tools are only trained on small datasets, and most are not tested on a wide range of review topic areas. This study presents two systematic review datasets compiled from more than 8600 systematic reviews and more than 540000 abstracts covering 51 research topic areas in health and medical research. These datasets are the largest of their kinds to date. We demonstrate their utility in training and evaluating language models for title and abstract screening. Our dataset includes detailed metadata of each review, including title, background, objectives and selection criteria. We demonstrated that a small language model trained on this dataset with additional metadata has excellent performance with an average recall above 95% and specificity over 70% across a wide range of review topic areas. Future research can build on our dataset to further improve the performance of fully automated tools for systematic review title and abstract screening.

## Highlights

### What is already known?

- There is an increasing number of fully automated tools for title and abstract screening, but their performance is suboptimal and varies substantially across review topic areas.
- The vast majority of these tools are not validated across a wide range of topic areas due to a lack of suitable datasets.

### What is new?

- This study compiles two datasets for systematic reviews, including metadata such as the title, background, objectives and selection criteria of each review. Our dataset is the largest of its kind to date, comprising more than 8600 systematic reviews and 540000 study abstracts across 51 health and medical research topic areas.
- We demonstrated that a small language model trained on this dataset achieves excellent performance with average recall above 95% and specificity over 70% across a wide range of review topic areas.

### Potential impact for RSM readers outside the authors’ field

- Systematic reviews are the gold standard of evidence synthesis in health and medical research. This study can contribute substantially to the development of fully automated tools for title and abstract screening, providing substantial benefits to the broader health and medical research community.

## 1 Background

In health and medical research, systematic reviews are the gold standard for evidence synthesis. The systematic review process involves several explicit and reproducible steps^1^: (1) specifying an a priori protocol that describe the scope and research question(s) to be reviewed; (2) systematically searching the literature to identify all potentially relevant publications; (3) distilling the large amount of potentially relevant publications, often in thousands or even tens of thousands, into a small set of publications (typically fewer than 50) that meet the criteria defined in an a priori specified protocol; and (4) extracting data from the included studies and synthesising the evidence.

Conducting systematic reviews is a painstaking process, with researchers often spending hundreds of hours searching and identifying relevant publications for evidence synthesis. The goal of a systematic review was to achieve total recall of literature corresponding to a specific research question. To minimise the risk of missing key publications, researchers start with a very broad search that often yields thousands of potential publications. It is not uncommon that over ten thousand publications are retrieved in this process, but on average, only 3% are relevant to the research question.^2^

Once the search is completed, at least two researchers then independently and manually examine titles and abstracts of each publication. This process is known as title and abstract screening. The researchers have to reach consensus on whether a publication is to be included for further full-text screening, a process where the researchers read the full publication carefully to determine if a publication is to be included in final evidence synthesis. Both title and abstract screening and full-text screening are error-prone and extremely time intensive. Recent studies showed that human screening error rates ranged from 6% to 21% depending on the research topics,^3^ and estimated that a systematic review on average takes a team of researchers 67 weeks to complete.^4^ The exponential growth of scientific literature and demand for systematic review in recent years has further escalated this challenge.

With advancements of artificial intelligence (AI) and natural language processing (NLP), researchers have been exploring the use of AI systems to automate the screening process.^5^^–^ ^8^ There are two main approaches: active learning (e.g.,^9^) and full automation (e.g.,^10^). Active learning adopts a “human-in-the-loop” approach and requires the researchers to label a small initial set of publication as “included” and “excluded” (or, “relevant” and “irrelevant” to the review scope). The system is then trained on this small dataset and ranks the remaining studies based on the probability of being included.^11^ Many of these systems will adopt a continued training approach, in which the researcher will then label another small subset from the remaining ranked studies. The system will continue to train on this additional data and re-rank the remaining studies. This is an iterative process and will be stopped using some pre-determined cut point or when the researchers subjectively determine that most relevant studies are identified. Such systems can often achieve a high level of recall (>0.95, a threshold that is considered as sufficient for systematic review automation), but their performance highly depends on the complexity of the systematic review’s research questions. This type of system often still requires substantial human involvement. For example, to achieve a 95% recall, the researchers may need to label over 90% of the publications,^12^ defeating the purpose of using automated AI systems.

On the other hand, fully automatic tools classify publications as included and excluded based on metadata of a systematic review such as the review title, objectives and selection criteria, or based on a few publications that were known to be included (i.e., using these studies as seed documents). While these systems hold the promise of greatly reducing researchers’ time in screening, their performance is in general lower than active learning systems. The acceptable goal for automated tools is to achieve at least 95% recall, but most automated screening tools have fallen short of this threshold.^6^ Recent research also evaluates the use of general purpose large language models such as GPT4 for automatic screening classification, but the performance has so far been disappointing.^13^

Furthermore, another key limitation identified in fully automatic tools is that most existing tools were trained and evaluated only on a small number of reviews (trained on fewer than 100 and evaluated on fewer than 10^6^). There is also a limited number of freely available datasets and these datasets often only have a small number of systematic reviews (<100; e.g.,^14^^,^ ^15^). This greatly limits the generalisability of these systems, as the performance of an AI classification system often deteriorates when it is applied to a domain that is different from training data.^16^ As demonstrated by another review of existing tools,^7^ screening performance varies substantially even when the same tool was applied to different research areas, with false-positive rate (identifying irrelevant publication as included) ranging from 1% to 81%.

As a result, despite active research and the availability of many screening automation tools, uptake of these tools has been slow and has been met with scepticism by the research community. For a tool to be widely adopted and used by applied researchers, its applicability and performance must be demonstrated across a wide range of areas.

The first aim of our study was therefore to address this critical lack of comprehensive training and evaluation data for automation of systematic review screening. We compiled a large systematic review dataset consisting of more than 8000 systematic reviews, with titles and abstracts of more than 500,000 individual publications from a wide range of research areas considered in the reviews. Metadata of each review, such as the review’s title, background, objectives and selection criteria, were included in the dataset. Each individual study is labelled as ‘included in the final evidence synthesis’, ‘included in title and abstract screening but excluded at full-text screening’ or ‘excluded during title and abstract screening’. This dataset is an essential resource for training a screening tool and evaluating its performance across research topic areas. It encompasses research ranging from allergy to mental health to medical research methodology.

The second aim was to demonstrate the utility of this dataset in training and evaluating AI models for full automation. Specifically, most existing tools were developed using limited metadata from a target systematic review. For example, to identify potentially relevant publications, some tools only used the title of a systematic review as the query (e.g.,^17^), and some only used the objective and selection criteria (e.g.,^18^). Other metadata such as the review’s background information often contains useful information that can help the screening process. Incorporating all the information about the review, including title of the review, background, objectives and selection criteria as the query in the training process can likely improve model performance. In practice, this information is readily available before the searching and screening process starts because the best practice for performing systematic review is to pre-register all this information in a protocol before the commencement of the review.^19^

Another limitation is that many systems were trained based on binary classification using data from a small number of existing systematic reviews. During training, a publication was either labelled as included or excluded. However, there could be more nuance in the data that can be used to improve the model performance. At the title and abstract screening stage, researchers often only excluded publications that were clearly irrelevant. Publications whose title and abstract were seemingly relevant or ambiguously written were often included for the next stage – full-text screening. The relevance of the title and abstract of these publications to the review was variable – some publications were eventually included in the evidence synthesis and some publications were excluded outright. This information about ambiguity can be exploited during training to potentially allow an AI model to learn richer representations that can improve classification. Our research question thus considers whether using additional metadata from individual systematic reviews and information about the title and abstract relevance for training can improve screening performance.

## 2 Methods

### 2.1 Dataset preparation

To support the development of an improved automated screening tool, we construct a large dataset of systematic reviews from the Cochrane Library. Specifically, we searched on 15 December 2023 to collect data on all systematic reviews published in the Cochrane Library.

Cochrane reviews are regarded by many as the gold standard of systematic reviews in health and medical research. Reviews published in the Cochrane Library undergo a rigorous review process and are scrutinised by peers and the Cochrane editorial team. Each Cochrane review’s abstract has a description of the background, objectives and selection criteria of individual studies (e.g., randomised controlled trials, cohort studies, etc), with a list of included studies, excluded studies and additional studies that are related to the review topic but were not included in the evidence synthesis. Many of the publications in these lists have hyperlink to their corresponding PubMed record or Google Scholar record, from which the abstracts of these publications can be retrieved.

The list of included, excluded and additional studies can be used to approximate how publications are screened during the screening process of a systematic review. A total of 8608 reviews and 544157 titles and abstracts were included in our dataset. It should be noted that the set of included, excluded and additional studies only approximate the set of publications that were included in the evidence synthesis, publications that passed through human title and abstract screening but excluded during full-text screening, and publications that were excluded during the title and abstract screening, respectively. The set of additional studies in each Cochrane review is (1) much smaller than the set of excluded publications in an actual systematic review and (2) these studies were still somewhat related to the review topic.

For our machine learning experiments, we split our dataset into 5 subsets. We withhold all reviews on the topic of heart and HIV as two test sets (N_review_ = 205 and N_review_ = 100, respectively). These two test sets allow evaluation of model performance on out-of-domain topic areas. For the remaining reviews, we randomly select 90% as the training set (N_review_ = 7458). For the remaining 10%, we randomly split it into a validation set (5%; N_review_ = 419) and a test set (5%; N_review_ = 426). A list of the 53 research areas covered by our dataset is presented in Supplementary Table 1.

Table 1 shows the descriptive statistics of our dataset. We have 259460 abstracts from publications excluded during the title and abstract screening, 191364 abstracts from publications excluded after full-text screening and 93333 abstracts from included publications.

**Table 1:** Descriptive statistics for the training set, validation set and the three test sets

			Excluded														
			during					Excluded									
	Number	Number	title and					after									
	of	of topic	abstract					full-text									
	reviews	areas	screening					screening					Included				
			Total	Mean	Median	Min	Max	Total	Mean	Median	Min	Max	Total	Mean	Median	Min	Max
Training set	7458	51	224622	30.2	26	1	1186	161003	23.7	12	1	682	81120	12.9	7	1	506
Validation set	419	49	12425	29.7	26	1	138	9922	25.4	11	1	273	4545	12.6	6	1	206
Test set – Random selection	426	51	13020	30.6	26	2	225	9448	24.2	12	1	209	4583	12.5	6	1	134
Test set – heart	205	1	7063	34.5	29	1	134	9500	47.5	19.5	1	530	2506	13.4	7	1	285
Test – HIV	100	1	2330	23.3	18	2	133	1491	17.1	11	1	91	579	7.2	4.5	1	38

The advantage of using this dataset for training is that it is a more ‘balanced’ dataset compared to an actual systematic review, as the vast majority of publications in the initial retrieved sets from an actual systematic review was publications that excluded during title and abstract screening. However, the disadvantage of using the Cochrane dataset is that it inflates the false-positive rate (false-negative rate was unaffected) during model evaluation. To evaluate the screening performance in actual systematic review, we compiled an additional test set by simulating the searching and screening process of 22 systematic reviews published in the Cochrane Library between 15 December 2023 and 15 May 2024. To do this, we followed the search strategies of these reviews to construct the set of initial publications for each review. Given the diverse search strategies used in different reviews, we only replicated the search in Medline, Embase and Cochrane Central.

The two datasets are available at https://data.mendeley.com/datasets/7sgmg89zb6/1 (DOI:10.17632/7sgmg89zb6.1).

### 2.2 Models

Two types of models were trained using our dataset. *Classification* models aim to classify a publication into one of three categories: included, excluded during the title and abstract screening, and excluded after full-text screening. These three categories were treated as mutually exclusive and independent categories, and information about the ordering of ‘relevance’ was not utilised. On the other hand, *Relevance* models involve predicting a score that indicates the degree of relevance of a publication to a given review scope. We set the relevance of an included publication as 1, those excluded during the title and abstract screening as 0, and those excluded after full-text screening as 0.5.

**Figure 1:**
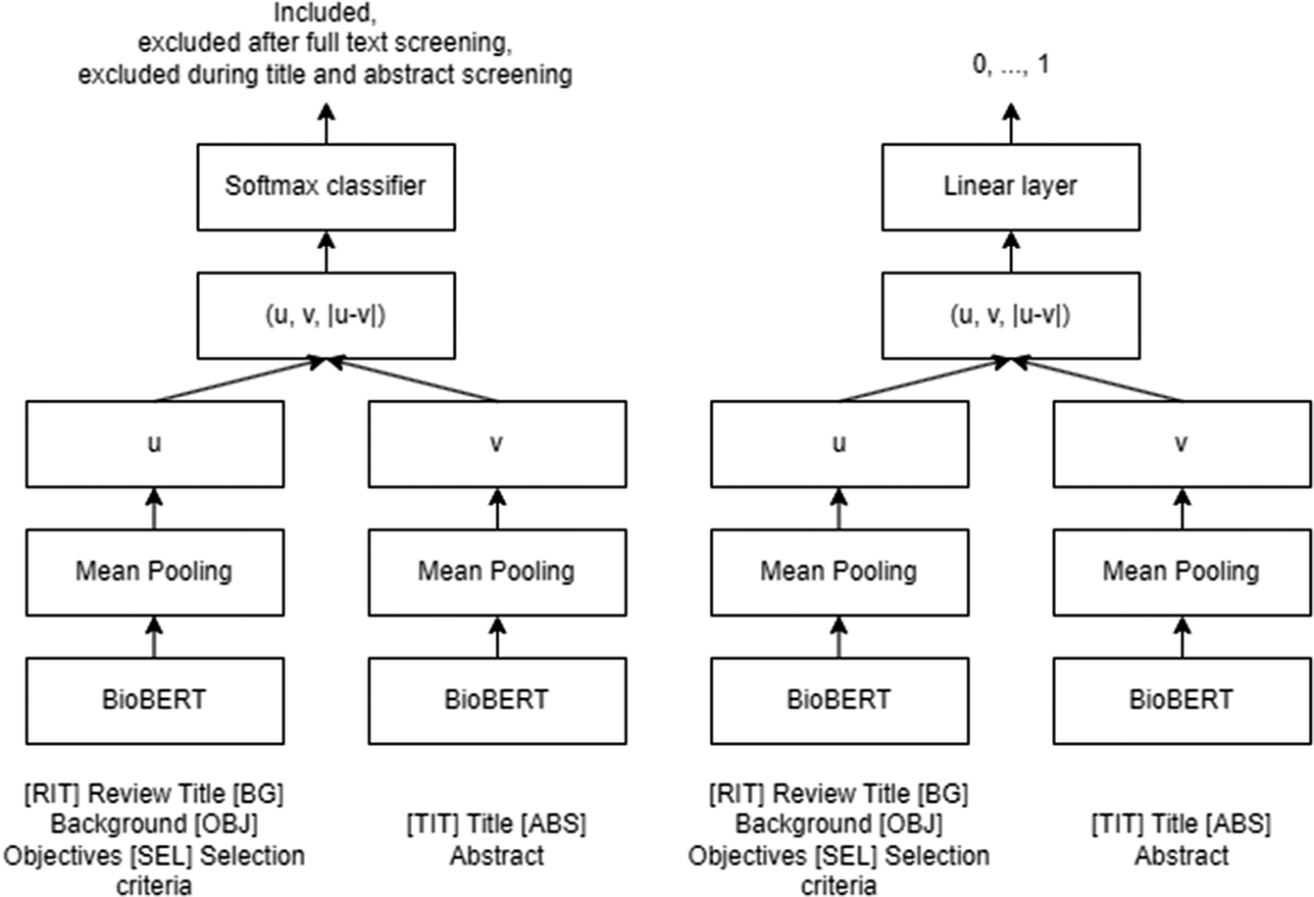
Classification model (left) and relevance model (right).

Both types of models are developed based on the Siamese Network using BioBERT^20^^,^ ^21^ (Figure 1), a pre-trained language model based on BERT (Bidirectional Encoder Representations from Transformer^22^) specifically optimised for biomedical text. Six custom tokens were added to the standard special tokens [CLS] and [SEP] in the vocabulary of the models to signify additional aspects, specifically the beginning of a review’s title *[RIT]*, Background *[BG]*, Objectives *[OBJ]*, Selection criteria *[SEL]*, and individual publication’s title *[TIT]* and abstract *[ABS]*.

The classification models were trained using a single softmax layer with a size of 1024 as the head of the models with cross-entropy loss. The relevance models were trained with a single linear layer of size 1024 as the head of the models to predict a relevance score of a publication from 0 to 1 with MSE loss. To allow comparison between these two types of models, we identified a ‘relevance cutoff score’ for classification that matches the false-negative rate of a corresponding classification model with the softmax layer using the validation set.

All models were trained on the training set for 1 epoch using the Adam optimiser with a learning rate of 0.00002, 200 warmup steps and a batch size of 32. Example model training codes are available at https://github.com/gckc123/systematic_review_paper_codes.

### 2.3 Evaluation metrics

There are two key evaluation metrics, false-positive rate and false-negative rate. False-positive rate is the percentage of publications that were eventually excluded in a systematic review but were determined as included by the model; false-negative rate is the percentage of publications that were eventually included in a systematic review but were determined as excluded by the model. We conduct two levels of analyses, publication level and review level. For publication-level analysis, we calculate the overall rate of false-negative and -positive rates across all publications for different models. Based on this analysis, we determine the optimal model (relevance model trained with title, background, objectives and selection criteria as the query; see Results section) for further review-level analysis. For review-level analysis, we calculate Precision@1, Precision@3 and Precision@5, the mean false-negative and false-positive rates across reviews, and the proportion of reviews that achieve total recall and 95% recall, respectively (0% and 5% false-negative). Precision@1, 3 and 5 are the proportion of ‘included’ publications in the top ranked 1, 3 and 5 publications based on relevance.

## 3 Results

Table 2 shows the results from the two types of models. The top panel shows the results from models trained using each review’s title, background, objective and selection criteria as the query; the bottom panel shows the results from models trained using only title, objective and selection criteria. The columns on the right-hand side show the results from the classification models that used a softmax layer to predict one of the three categories: *included, excluded after full-text screening* and *excluded during title and abstract screening*. The latter two categories were combined as ‘Excluded’ when calculating the false-positive and false-negative rates. The columns on the left-hand side show the results from the relevance models that predicts a relevance score from 0 to 1. For comparison, we identified a cutoff score using the validation set that matched the false-negative rate of the classification model and using the cutoff to classify a publication should be included or excluded. Test of proportion was conducted to compare the sensitivity and specificity across models.

**Table 2:** Model performance of the 4 models. False negatives are **bold** and false positives are underlined

	Relevance model				Classification model			
	Model trained using title, background,				Model trained using title, background,			
	objective, Selection criteria				objective, Selection criteria			
	Model decision				Model decision			
	Exclude		Include		Exclude		Include	
Validation set	N	%	N	%	N	%	N	%
Excluded during title and abstract screening	11756	94.62%	669	5.38%	11392	91.69%	1033	8.31%
Excluding after full-text screening	2760	27.82%	7162	72.18%	1354	13.65%	8568	86.35%
Included	**114**	**2.51%**	4431	97.49%	**114**	**2.51%**	4431	97.49%
**Test set (random)**								
Excluded during title and abstract screening	12166	93.44%	854	6.56%	11666	89.60%	1354	10.40%
Excluding after full-text screening	2416	25.57%	7032	74.43%	1255	13.28%	8193	86.72%
Included	**158**	**3.45%**	4425	96.55%	**100**	**2.18%**	4483	97.82%
**Test set (heart)**								
Excluded during title and abstract screening	6583	93.20%	480	6.80%	6177	87.46%	886	12.54%
Excluding after full-text screening	2314	24.36%	7186	75.64%	1342	14.13%	8158	85.87%
Included	**78**	**3.11%**	2428	96.89%	**80**	**3.19%**	2426	96.81%
**Test set (HIV)**								
Excluded during title and abstract screening	1993	85.54%	337	14.46%	1905	81.76%	425	18.24%
Excluding after full-text screening	608	40.78%	883	59.22%	431	28.91%	1060	71.09%
Included	**52**	**8.98%**	527	91.02%	**42**	**7.25%**	537	92.75%
**Validation set**	N	%	N	%	N	%	N	%
Excluded during title and abstract screening	11402	91.77%	1023	8.23%	11417	91.89%	1008	8.11%
Excluding after full-text screening	2002	20.18%	7920	79.82%	1460	14.71%	8462	85.29%
Included	**128**	**2.82%**	4417	97.18%	**130**	**2.86%**	4415	97.14%
**Test set (random)**								
Excluded during title and abstract screening	11686	89.75%	1334	10.25%	11670	89.63%	1350	10.37%
Excluding after full-text screening	1738	18.40%	7710	81.60%	1270	13.44%	8178	86.56%
Included	**99**	**2.16%**	4484	97.84%	**92**	**2.01%**	4491	97.99%
**Test set (heart)**								
Excluded during title and abstract screening	6388	90.44%	675	9.56%	6194	87.70%	869	12.30%
Excluding after full-text screening	1913	20.14%	7587	79.86%	1386	14.59%	8114	85.41%
Included	**76**	**3.03%**	2430	96.97%	**75**	**2.99%**	2431	97.01%
**Test set (HIV)**								
Excluded during title and abstract screening	1955	83.91%	375	16.09%	1933	82.96%	397	17.04%
Excluding after full-text screening	537	36.02%	954	63.98%	458	30.72%	1033	69.28%
Included	**48**	**8.29%**	531	91.71%	**41**	**7.08%**	538	92.92%

Overall, at a similar level of false negative, the relevance models have a significantly lower level of false positive in all three test sets compared to the classification models (*p* < .05). In other words, at a similar level of sensitivity, the relevance models have a higher level of specificity. This suggests that training models using information about the relevance of the publications can improve model performance.

With regard to including review’s background in addition to the title, objective and selection criteria as the query, the performance from the classification models trained with and without background are similar. However, for relevance models, the model trained with review’s background has lower false positives at a similar level of false negatives for the validation and all three test sets. Supplementary Figure 1 and 2 show the receiver operating characteristics curve (ROC curve) from the relevant models with and without background, and the area under the curve from the model with background was all higher than the corresponding models without, further demonstrating that including review’s background can improve model performance.

Overall, all models have a false-negative rate below the 5% threshold in the validation set, random test set and Heart test set. The false-negative rate for the HIV test set falls short of this threshold and is at around 8%.

These results were publication-level analysis and demonstrated that the relevance model trained with background in general has lower false positives and false negatives. In Table 3, we present the results from a review-level analysis using our best performing model – the relevance model trained using review’s title, background, objectives and selection criteria as query. For example, Precision@1 for the validation set and test sets ranged from 0.65 to 0.77, indicating that in 65% to 77% of the reviews, the highest ranked publication is a publication that should be included. The false-negative rates for the validation set, random test set and heart test set were all below 5%. However, similar to the publication-level analysis, the false-negative rate for the HIV test set was 8%. The false-positive rate ranged from 67% to 72%; however, it should be noted that these are inflated estimates as per explanation in the previous section. Overall, around in around 80% of the review, total recall was achieved.

**Table 3:** Review-level analysis on the relevance model

				False negative		Total recall	95% recall	False positive	
	Precision@1	Precision@3	Precision@5	Mean	SD	%	%	Mean	SD
Validation set	0.772	0.719	0.723	0.04	0.135	0.8116	0.8587	0.7087	0.2947
Test set – Random	0.724	0.718	0.711	0.039	0.119	0.812	0.7474	0.7243	0.2826
Test set – Heart	0.652	0.605	0.589	0.049	0.147	0.7957	0.8387	0.708	0.269
Test set – HIV	0.69	0.712	0.675	0.08	0.187	0.788	0.8125	0.6694	0.262

**Figure 2:**
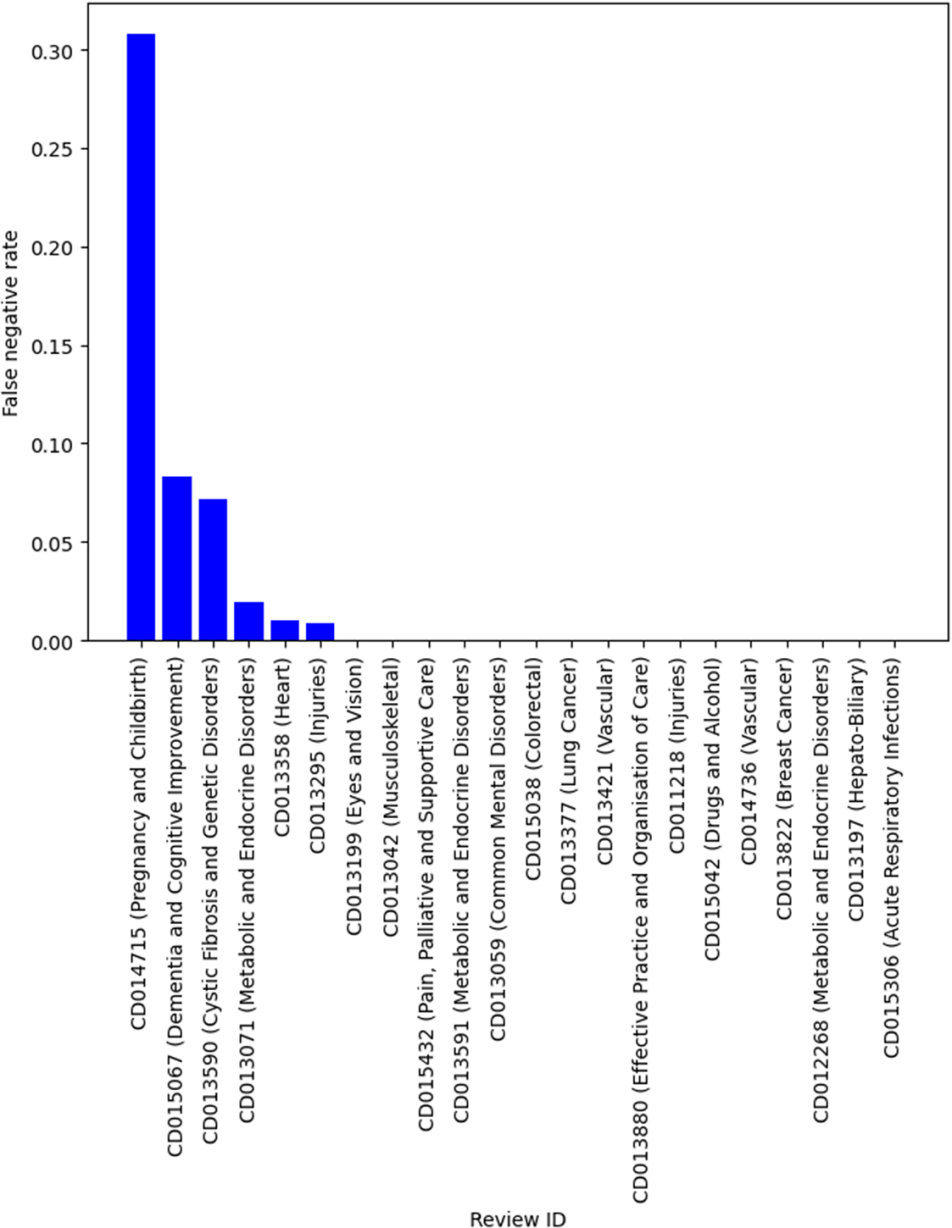
False-negative rate by reviews.

**Figure 3:**
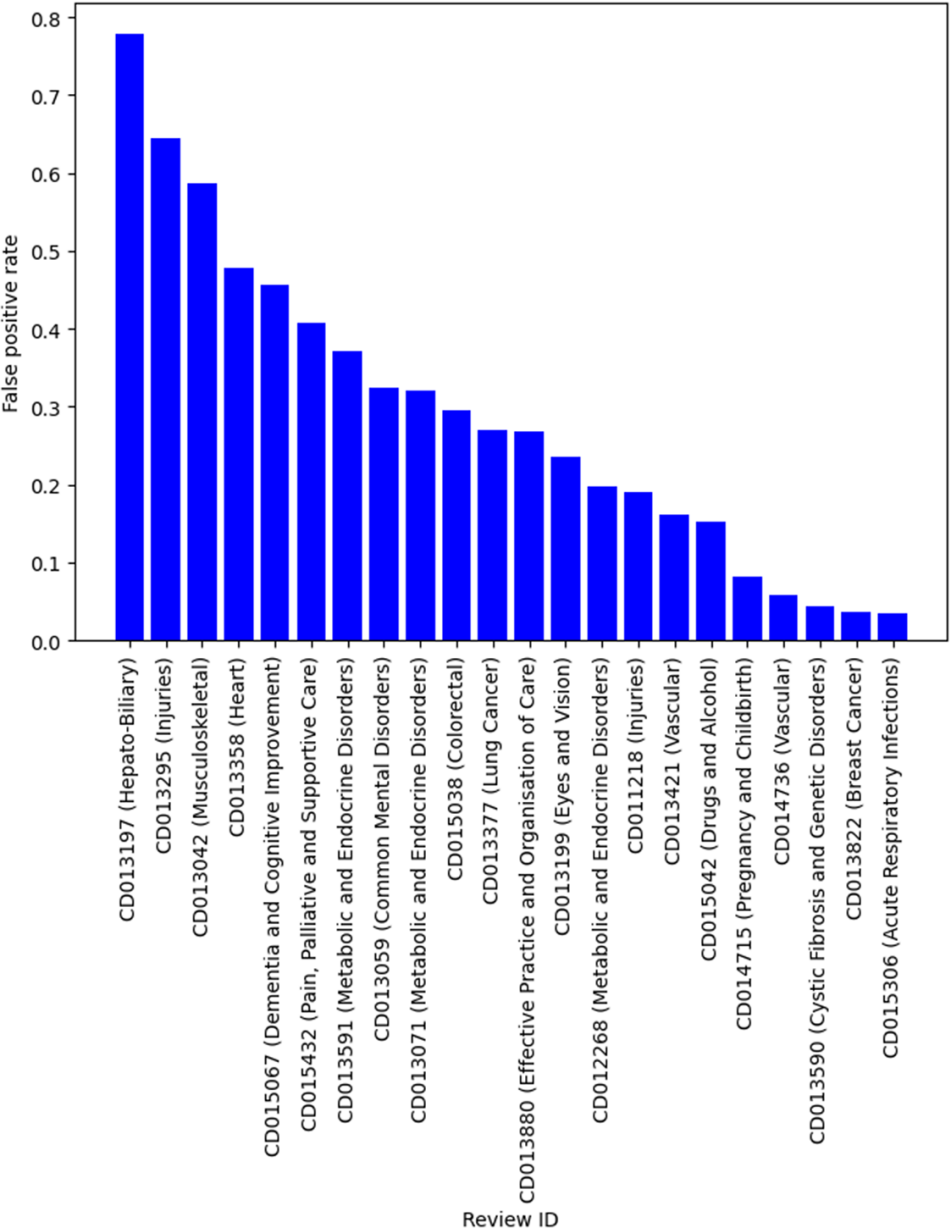
False-positive rate by reviews.

Table 4 shows the descriptive statistics of the smaller systematic review dataset that was compiled by simulating the search and screening process based on the search strategies documented in the original reviews. There were 22 reviews in this dataset, with over 142000 titles and abstracts from individual publications. This is a much more imbalanced dataset with the vast majority of publications being irrelevant and should be excluded during title and abstract screening. Similarly, we used the relevance model trained using title, background, objectives and selection criteria, on this dataset to provide better estimates of the performance (such as specificity) in practice. Although the sample size was small, it has a similar recall compared to the results in Table 3. For example, in 19 of 22 reviews, 95% recall was achieved. Two reviews have a false-negative rate over 5% but was still under 10%. There was one outlier with a very high false-negative rate (30.82%). This was a review of a diagnostic test, which has been known to be difficult to screen even for human reviewers because of the large variation in study design. The false-positive rate on average was low (mean and median both below 30%). However, there was large variability, with the false-positive rate ranging from 3% to 78%. Figures 2 and 3 show the false-negative and -positive rates for each review, respectively.

**Table 4:** Descriptive statistics of a small systematic review data by simulating manual search

Descriptive statistics	
Number of reviews	22
Total number of retrieved publications	142504
Number of retrieved publications per review	
Mean	6477
Median	3097
Min	690
Max	49162
Analysis results	
False-positive rate (specificity)	
Mean	29.06% (70.94%)
Median	26.88% (73.12%)
Min	3.48% (96.52%)
Max	77.94% (22.06%)
Maximum false-negative rate	30.82%
Number of reviews with total recall	16 (out of 22; 72.73%)
Number of reviews with 95% recall	19 (out of 22; 86.36%)

## 4 Discussion

There are some existing tools for systematic review automation, many of which claim to be able to perform title and abstract screening. There is relatively large variability in performance between these tools, and even within the tool when it is applied to different systematic review topics. For example, recent review has shown that the false-positive rate can range from 1% to 81% even when the same tool was applied on different research areas.^7^ Many of these tools are only trained on data from a small number of reviews and the performance of these tools is also not validated across a wide range of topics. As a result, these tools have not been widely adopted.

In this study, we compiled a large systematic review dataset for training and evaluating tools that automate title and abstract screening. We demonstrated the clear utility of this dataset. A recent large review of automated title and abstract screening tools by Feng, Liang, Zhang, Chen, Wang, Huang, Sun, Liu, Zhu and Pan^6^ shows that the combined recall and specificity of existing tools were 92.8% and 64.7%, respectively. We trained several small models using this dataset, and our results demonstrated our model achieved higher recall on average (>95%). At the review level, total recall was achieved in most reviews. Furthermore, using a more realistic systematic review dataset created by simulating actual manual publication search, we demonstrate that our small models also achieved higher specificity (> 70%).

There are two key findings from the empirical experiments in this study. First, we demonstrated that training models with richer information as features, including using the review’s title, background, objectives and selection criteria, improves model performance. At a fixed recall level, the false-positive rate was lower in models trained with all 4 pieces of information, compared to models trained without the background section. Second, we demonstrated the value of utilising information derived from those publications in three distinct groups—those that were excluded outright at title and abstract screening, those that were excluded after full-text screening and those that were included in the final evidence synthesis. Many systems are trained using limited metadata of the review as a query, for example, only the title,^17^ or the title, objectives and selection criteria,^18^ or using a seed publication.^23^ We have demonstrated that the background of a review provides important information about the review. Such information is often available before researchers start the literature search process because the best practice of systematic review is to pre-register the review protocol in open access platforms such as PROSPERO.

Although the performance of our models is good on average, there is substantial variability in the performance. For a complex research question or topic, such as a review of diagnostic test efficacy, the performance of our models is not sufficient to be used in practice. Reviews on diagnostic tests of a medical condition are well-known to be difficult to screen, because unlike reviews of interventions for a medical condition, the design of diagnostic tests can vary substantially between studies. For example, evaluation of a diagnostic test can be based on the prospective cohort study, retrospective case review, laboratory and experimental study. For a medical condition, it is not uncommon that a wide range of diagnostic tests from urine tests to skin and blood tests are available. In our small but more realistic dataset, our model achieved good recall in all but one review which focuses on a diagnostic accuracy of ultrasound screening for foetal structural abnormalities. In this review, 30% of relevant publications were excluded. The suboptimal performance for reviews on diagnostic tests is likely due to the model having an insufficient understanding of the study abstract and/or the review’s objective and selection criteria to accurately determine whether a study should be included.

Future research can build on our dataset to further improve the performance of automated title and abstract screening systems. For example, we used BioBERT in this study, which has a small context window of only 512 tokens. Many of the titles and abstracts in our dataset exceed this context window and were thus truncated. Further, the scaling laws of language models demonstrates that model performance increases when the model size increases.^24^ Since BioBERT is relatively small compared to the state-of-art language models, using larger models with larger context windows will likely further improve performance. Recent research has demonstrated that larger models generally perform much better in tasks requiring higher levels of natural language understanding.^25^ Thus, the suboptimal performance of BioBERT in reviews on diagnostic tests may be improved by using larger models. There is also emerging research that uses general generative large language models, such as GPT-4, for title and abstract screening, but their performance is in general worse than fine-tuned smaller models.^13^ With quantisation and low-rank adaptation,^26^ further pre-training a larger model (e.g., Llama-3 8B) first on biomedical text and then fine-tuning it using our dataset is likely to reduce both false-positive and -negative rates further. Another possible avenue to improve model performance is to train a mixture-of-experts (MoE)^27^ model based on BioBERT. An MoE model contains several versions of feed-forward neural networks in the transformer architecture to act as different “Experts” to process the input. In the context of systematic review screening, such a model can utilise a gating mechanism that based on the review’s metadata and send the corresponding information to a corresponding “Expert” for processing. This type of architecture has been shown to enhance language model performance in a range of language tasks, with performance on par with much larger dense language models.^27^

## 5 Conclusion

We have demonstrated that the utility of using a large systematic review dataset for training and evaluating automated title and abstract screening systems for systematic review. Our results are promising and our simple models on average have lower false-positive and -negative rates than most existing systems. We have demonstrated the value of finer-grained consideration of the relevancy of publications to a review question, as well as the use of more background context about the review to support improved screening. Future research can build upon the dataset we compiled in this study to further improve the performance of automated title and abstract screening systems.

## Supporting information

Chan et al. supplementary material Chan et al. supplementary material

## Data Availability

The data is available at DOI: 10.17632/7sgmg89zb6.1.

## Supplementary material

The supplementary material for this article can be found at https://doi.org/10.1017/rsm.2025.1.
