# Supplementary material for: A comprehensive systematic review dataset is a rich resource for training and evaluation of AI systems for title and abstract screening
Source: Res Synth Methods. 2025 Mar 7;16(2):308–22. doi: 10.1017/rsm.2025.1 (PMC12527522; doi:10.1017/rsm.2025.1)

Supplementary Material.

Supplementary Table 1. List of topic area in our dataset.

Cochrane Review Group Code		
Acute Respiratory Infections	Gut	Oral Health
Airways	Gynaecological, Neuro-oncology and Orphan Cancer	Pain, Palliative and Supportive Care
Anaesthesia	Gynaecology and Fertility	Pregnancy and Childbirth
Back and Neck	Haematology	Public Health
Bone, Joint and Muscle Trauma	Heart	Schizophrenia
Breast Cancer	Hepato-Biliary	Sexually Transmitted Infections
Childhood Cancer	HIV	Skin
Colorectal	Hypertension	Stroke
Common Mental Disorders	Incontinence	Tobacco Addiction
Consumers and Communication	Infectious Diseases	Urology
Cystic Fibrosis and Genetic Disorders	Injuries	Vascular
Dementia and Cognitive Improvement	Kidney and Transplant	Work
Developmental, Psychosocial and Learning Problems	Lung Cancer	Wounds
Drugs and Alcohol	Metabolic and Endocrine Disorders	
Effective Practice and Organisation of Care	Methodology	
Emergency and Critical Care	Movement Disorders	
ENT	Multiple Sclerosis and Rare Diseases of the CNS	
Epilepsy	Musculoskeletal	
Eyes and Vision	Neonatal	
Fertility Regulation	Neuromuscular	

Supplementary Figure 1. ROC Curve for relevance model trained using review's title, background, objectives and selection criteria as query. Top left: Validation set; Top right: random test set; Bottom left: Test set - Heart; Bottom right: Test set - HIV.

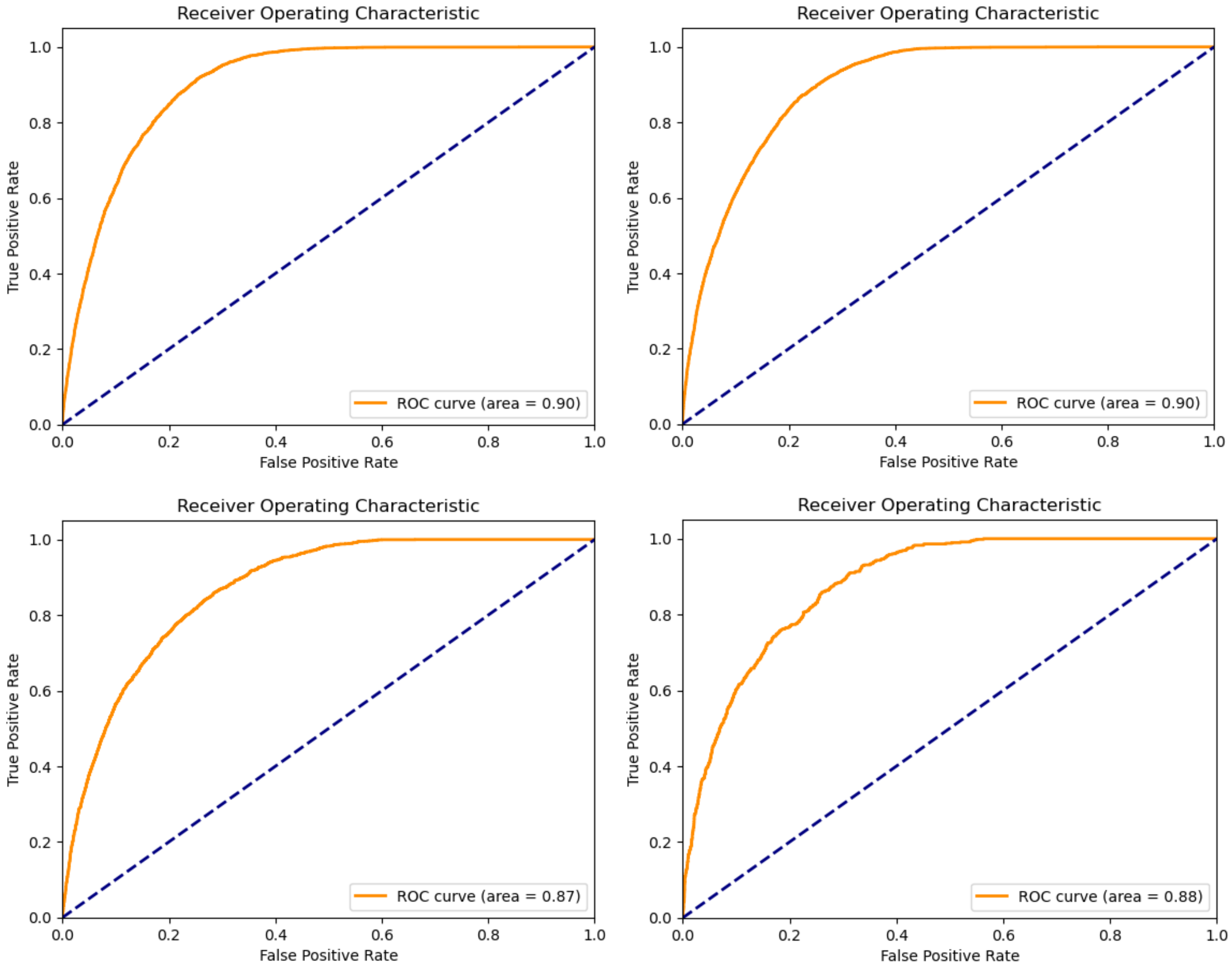

Supplementary Figure 2. ROC Curve for relevance model trained using review's title, objectives and selection criteria as query. Top left: Validation set; Top right: random test set; Bottom left: Test set - Heart; Bottom right: Test set - HIV.

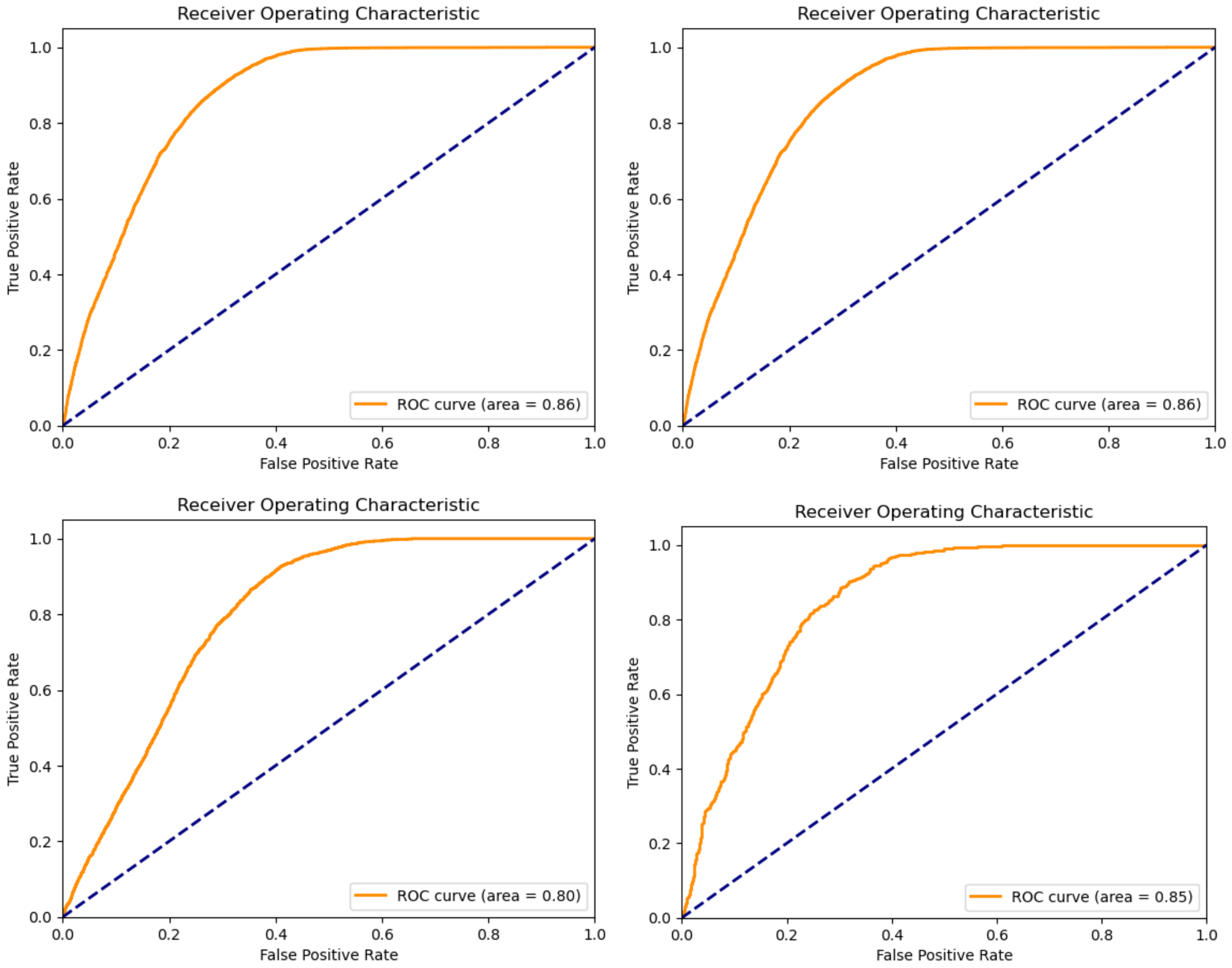

Supplement: Chan et al. supplementary material [file S1759287925000018sup001.pdf]
